# What are the age-related factors linked to aseptic revisions in constrained and unconstrained TKA as well as UKA? A register-based study from the German arthroplasty registry (EPRD)

**DOI:** 10.1007/s00402-024-05550-9

**Published:** 2024-09-11

**Authors:** Josina Straub, Dominik Szymski, Nike Walter, Yinan Wu, Oliver Melsheimer, Alexander Grimberg, Volker Alt, Arnd Steinbrueck, Markus Rupp

**Affiliations:** 1 Department of Trauma Surgery, University Medical Centre Regensburg, Franz-Josef-Strauss Allee 11, 93053 Regensburg, Germany; 2 Deutsches Endoprothesenregister gGmbH (EPRD), Berlin, Germany; 3 Orthopädisch Chirurgisches Kompetenzzentrum Augsburg (OCKA), Augsburg, Germany

**Keywords:** Knee arthroplasty, Aseptic revision, Register study, Unicondylar, Age-related risk factors

## Abstract

**Purpose:**

The implantation rate of total knee arthroplasties (TKA) is continuously growing. Aseptic problems are a major cause of revision. The aim of the following study was to determinate the incidence of aseptic revisions in primary knee arthroplasty as well as aseptic revision rates and influencing factors according to the patients’ age and type of procedure.

**Methods:**

Data collection was performed using the German Arthroplasty Registry. Influencing factors were analyzed according to the patients’ age and type of procedure. Risk factors were calculated using multiple Log-rank test with the Holm’s method. Incidence and comparison of aseptic revisions according to the patients’ age and type of procedure were analyzed using Kaplan-Meier-estimates. Cox regression was applied to calculate the hazard ratio.

**Results:**

Overall, 300,998 knee arthroplasties with 254,144 (84.4%) unconstrained TKA, 9,993 (3.3%) constrained TKA and 36,861 (12.3%) unicondylar knee arthroplasties (UKA) were analyzed. Patients younger than 65 years suffered a significantly higher aseptic revision rate than older patients (*p* < 0.0001). After one year, a revision rate of 1.1% was recorded for patients 65–74 years, 1.6% for patients under 65 years, and 1.3% for patients beyond 74 years. After seven years, patients younger than 65 years sustained in 5.0%, patients 65–74 years in 2.9% and patients beyond 74 years in 2.4% revision. In unconstrained TKA, an increased Elixhauser-score (HR = 1,75; HR = 1,54; HR = 1,7; *p* < 0,001) was a risk factor regardless the age. A TKA volume of 101–250 regardless the age (HR = 0,66; HR = 0,69; HR = 0,79) and > 250 under 75 years (< 65: HR = 0,72; 65–74: HR = 0,78; *p* = 0,001) were protective for aseptic revision. In UKA, male gender (HR = 0,81; HR = 0,72; HR = 0,57; *p* < 0,001), a UKA volume ≥ 51 for patients under 75 years (< 65: HR = 0,62; 65–74: HR = 0,59; *p* = 0,003) as well as cemented UKA for patients younger than 75 years (< 65: HR = 0,37; 65–74: HR = 0,37; *p* < 0,001) were detected as preventive factors.

**Conclusion:**

A significant increased rate of aseptic revisions was reported for patients younger than 65 years compared to older patients. An increased Elixhauser score was a risk factor, whereas male and a high volume of performed UKA or TKA could be identified as preventive factors.

**Level of evidence:**

III, cohort study.

## Introduction

Knee arthroplasty is one of the most common performed orthopedic procedures worldwide and is the preferred treatment for patients with end-stage osteoarthritis of the knee [1]. Of all total knee arthroplasty (TKA) procedures, unconstrained TKA accounts for 80–90% of cases for patients with osteoarthritis of the knee [2]. By 2040, the implantation rate of TKA in Germany will increase from 245 TKA’s per 100.000 inhabitants in the year 2016 to 379 [3]. A total of 1,485,482 knee arthroplasty procedures were performed in the USA between 2012 and 2021 [4]. Moreover, primary TKA use in the United States increased from 38.4% in the period 2001–2005 to 42.7% in 2006–2010 for patients 65 years and younger [5]. In the United States, about 72,100 revisions of TKA were carried out in 2014 [6]. By 2030, it is anticipated that this number will increase according to the Poisson regression model 182% and according to the linear model 78%, respectively [6]. Unicondylar knee arthroplasty (UKA) constituted 2.9% of all primary knee arthroplasties reported to the American Joint Replacement Register (AJRR) in 2017 and increased to 4.2% in 2021 [1]. However, only 5–10% of knee arthroplasty procedures are performed with UKA. Moreover, UKA is associated with a threefold increase in revision rates when compared to TKA [20]. A total of 46.3% additional procedures were performed for UKA revisions over ten years [7]. Revision surgery is anticipated to be necessary for a higher percentage of younger individuals as the number of younger patients undergoing TKA rises [8]. Reasons for revisions can either be septic or aseptic reasons and the most frequent reasons for revisions were septic causes, aseptic loosening and wear [9–11]. The analysis of failed TKA in the United States healthcare system demonstrated infection being the most common reason for revision (20.4%), followed by aseptic loosening (20.3%) [9]. Rates and influencing factors of aseptic revisions after primary knee arthroplasty according to the different patients’ age are an issue with only a limited data available for unconstrained and constrained TKA as well as UKA.

Therefore, the aim of the present study was to (1) analyse the incidence of aseptic revisions in unconstrained and constrained primary TKA as well as UKA. In addition, (2) an analyses of aseptic revision rates as well as influencing factors according to the patients’ age and type of procedure was performed.

## Materials and methods

### Data collection

**Fig. 1:**
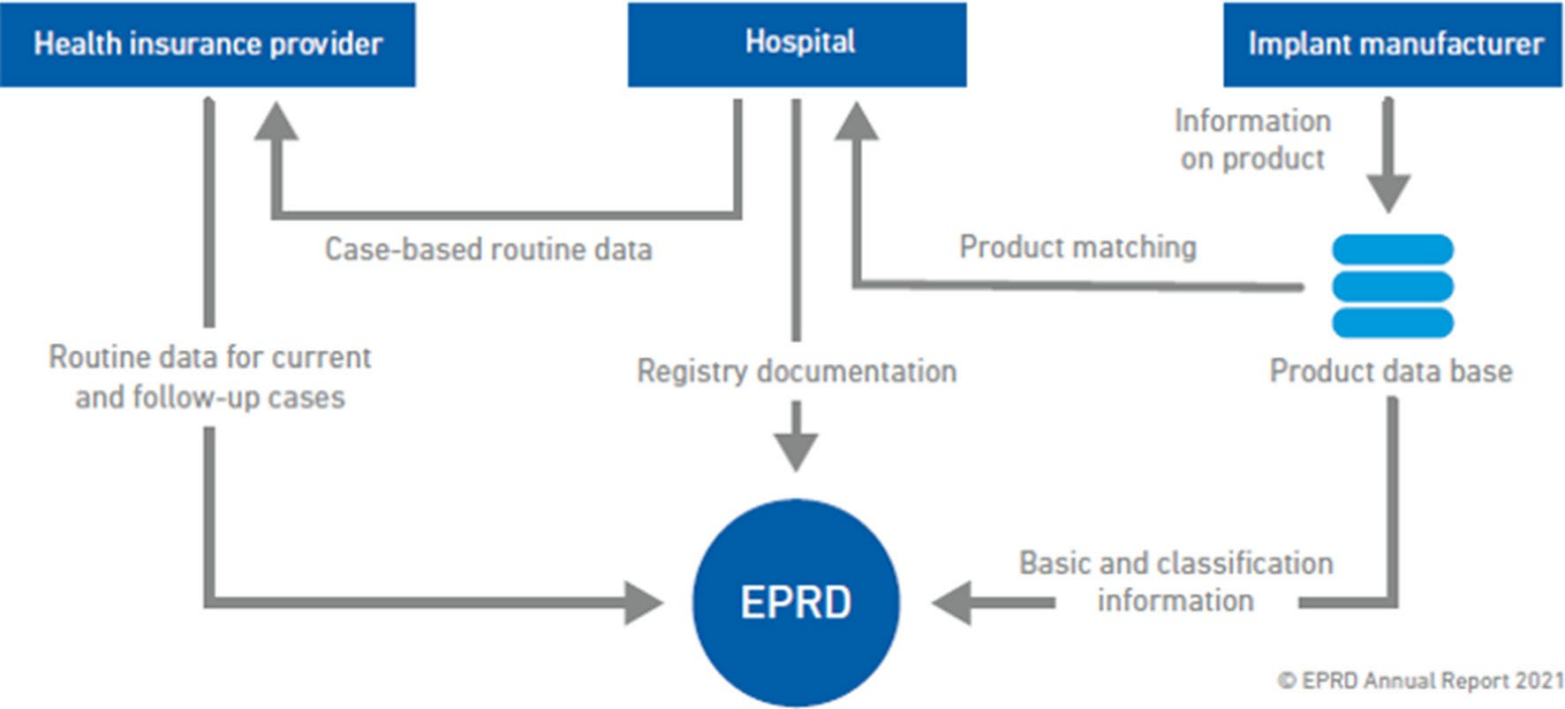
The flow of data from hospitals, health insurance and implant manufacturers to the EPRD

The study was approved by the Ethics Committee of the University of Kiel (ID: D473/11) and conducted according to the Declaration of Helsinki. This research analyzes data from the “German Arthroplasty Registry” (EPRD) to investigate aseptic revisions of constrained and unconstrained TKA as well as UKA in patients with primary osteoarthritis of the knee. Since 2012, the German Medical Technology Association (BVMed), the statutory health insurance funds (AOK Bundesverband GbR, Verband der Ersatzkassen e.V vdek), and several participating hospitals have collaborated to document arthroplasty implants in Germany through the “German Arthroplasty Registry” (EPRD). Over 2 million procedures are included in the registry and approximately 70% of all hip and knee arthroplasties performed in Germany are covered in the registry by 2022 [12]. Cross-validation of data provided by the surgeons is performed by inclusion of two participating health insurance associations (AOK-B, vdek), which approximately covers hereby 65% of the German population. Surgical revisions registered in the EPRD are followed up based on insurance billing data, even if performed in a hospital not participating in the arthroplasty registry. Except for medical procedures performed outside of Germany, this system ensures nearly perfect tracking of patients insured by the insurance of these corporations [14] (See Fig 1).

Diagnoses and procedures were categorized and identified using the German versions of the 10th International Classification of Diseases (ICD-10), the “Operation and Procedure Code” (OPS) 301 system, and the International Classification of Procedures in Medicine (ICPM).

### Patients

The patients receiving TKA or UKA following primary osteoarthritis of the knee as the primary diagnosis between November 2012 and September 2022 are included in the current analysis of the German Arthroplasty Registry (EPRD) (ICD-10: M17.0-, M17.1). Subpopulations of patients with UKA as well as constrained and unconstrained TKA were identified and a separation into three age groups was carried out for the analysis of influencing factors according to the patients’ age and type of prothesis. The three age groups were defined as ≤ 64 years, 65–74 years and ≥ 75 years. The registry contained information on patient characteristics such as age, sex, Body-Mass-Index (BMI), American Society of Anesthesiologists risk score (ASA), Elixhauser-Comorbidity Score and hospital-related parameters including TKA volume. The Elixhauser score is an index that combines a number of comorbidities from various organ systems and entities [13]. Coded comorbidities in the initial hospital stay during primary implantation of the arthroplasty were the basis for the calculation of the Elixhauser-Score. The National Joint Registry (NJR) and EPRD common product libraries’ classification data were used to evaluate the implant used after surgery and determine whether to employ unconstrained or constrained TKA. Revision rate was determined through search of the ICD-10 code for aseptic revision (T84.5) in the registry and registration of revision causes by the surgeons. The method and side of interest were thoroughly registered in the “Operation and Procedure Code” (OPS-Codes) analysis. Data provided by the registration of surgeons was cross-validated by analysis of insurance data. Patients without a clear history of used material, with a follow-up of less than a year, without treatment for primary gonarthritis as the primary diagnosis, and with an implantation of a special implant as well as individualized implants were not included in the analysis.

### Statistical analysis

According to the various patients’ age in Germany, the data were investigated to determine the rates and influencing factors of aseptic revisions in both constrained and unconstrained TKA as well as UKA. The statistical analysis was done using the statistical package R (R Foundation for Statistical Computing, version 4.2, Vienna, Austria). Categorical variables were presented in terms of frequency and percentage. Descriptive Statistics were calculated for the unconstrained and constrained TKA as well as UKA. Continuous variables are presented in mean and standard deviation, categorical variables in number of observations and frequency. The corrected Multiple Log-rank test with Holm’s technique was used to compare the three type of prothesis and the three various age groups. Cumulative incidences for the aseptic revision endpoint as well as cumulative incidences for the aseptic revision endpoint according to the different groups of ages were computed by using Kaplan-Meier estimates. A Cox proportional-hazard model was used to evaluate the effects of constrained and unconstrained TKA as well as UKA according to the various age groups with adjusted risk factors. However, the assumption of constant proportional hazards was violated in case of unconstrained TKA and UKA by the confounding variables, including the weighted Elixhauser score, age group, and BMI. Therefore, we split the time axis at six months after the operation. The significance level was defined at 5%.

## Results

In the “German Arthroplasty Registry” (EPRD) 396,284 primary arthroplasty knee procedures were identified. After exclusion of patients not matching the inclusion criteria 300,998 patients were included into the final analysis. 254,144 (84.4%) patients received an unconstrained TKA, 9,993 (3.3%) a constrained TKA and 36,861 (12.3%) an UKA for treatment of primary osteoarthritis of the knee. Across all types of procedures, female patients received knee protheses most frequently. Female patients accounted for 66% of unconstrained TKA, 78% of constrained TKA, and 56% of UKA. The Elixhauser score weighted in numeric was calculated 0.9 for unconstrained TKA, 2.3 for constrained TKA and 0.2 for UKA. Patient characteristics of unconstrained and constrained TKA as well as UKA are summarized in Table 1.

**Table 1:** Anthropometric data on patient collective

Characteristic	TKA unconstrained, *N* = 254,144	TKA constrained, *N* = 9,993	UKA, *N* = 36,861
**Age**			
< 65	78,197 (30,8%)	1,926 (19,3%)	19,316 (52,4%)
65–74	86,654 (34,1%)	2,859 (28,6%)	10,645 (28,9%)
≥ 75	89,293 (35,1%)	5,208 (52,1%)	6,900 (18,7%)
**Sex**			
Female	168,851 (66%)	7,796 (78%)	20,796 (56%)
Male	85,293 (34%)	2,197 (22%)	16,065 (44%)
**BMI (kg/m²)**			
Underweight (< 18,5)	297 (0,1%)	39 (0,3%)	41 (0,1%)
Normal (18,5–24,9)	22,946 (9%)	1,394 (14%)	3,471 (9,4%)
Pre-obese (25–29,9)	58,694 (23,1%)	2,275 (22,8%)	9,336 (25,3%)
Obesity grade I (30–34,9)	49,958 (19,7%)	1,665 (16,6%)	7,285 (19,7%)
Obesity grade II (35–39,9)	25,188 (9,9%)	865 (8,7%)	3231 (8,8%)
Obesity grade III (> 40)	14,205 (5,6%)	625 (6,3%)	1,309 (3,6%)
Unknown	82,856 (32,6%)	3,130 (31,3%)	12,188 (33,1%)
**ASA**			
1	6,038 (2,4%)	212 (2,1%)	1,235 (3,4%)
2	30,288 (11,9%)	1,004 (10%)	5,184 (14,1%)
≥ 3	17,569 (6,9%)	896 (9,1%)	1,830 (5%)
Unknown	200,249 (78,8%)	7,881 (78,8%)	28,612 (77,5%)
**Elixhauser score**			
< 0	59,003 (23%)	1,844 (18%)	8,460 (23%)
0	118,592 (47%)	3,882 (39%)	20,607 (56%)
1–4	27,432 (11%)	1,150 (12%)	3,308 (9.0%)
≥ 5	49,117 (19%)	3,117 (31%)	4,486 (12%)

Medical centers performing > 250 TKA procedures annually completed 40.8% of unconstrained TKA, while medical centers with a TKA volume of 101–250 performed with 38.8% the majority of the constrained TKA. With 52% the majority of UKA were completed in a center operating more than 50 UKA annually. Characteristics of the treating hospital are summarized in Table 2.

**Table 2:** Hospital characteristics of hospitals performing TKA and UKA implantations of the included patient collective

Characteristic	TKA unconstrained, *N* = 254,144	TKA constrained, *N* = 9,993	UKA, *N* = 36,861
**TKA implantation volume**			
0-100	54,541 (21,5%)	2,620 (26,2%)	
101–250	89,092 (35,1%)	3,876 (38,8%)	
> 250	103,567 (40,8%)	3202 (32%)	
Unknown	6,944 (2,6%)	295 (3%)	
**UKA implantation volume**			
0–10			2,090 (5,7%)
11–50			13,210 (35,8%)
> 50			19,191 (52%)
Unknown			2,370 (6,4%)

^1^Mean (SD); n (%);

**Fig. 2:**
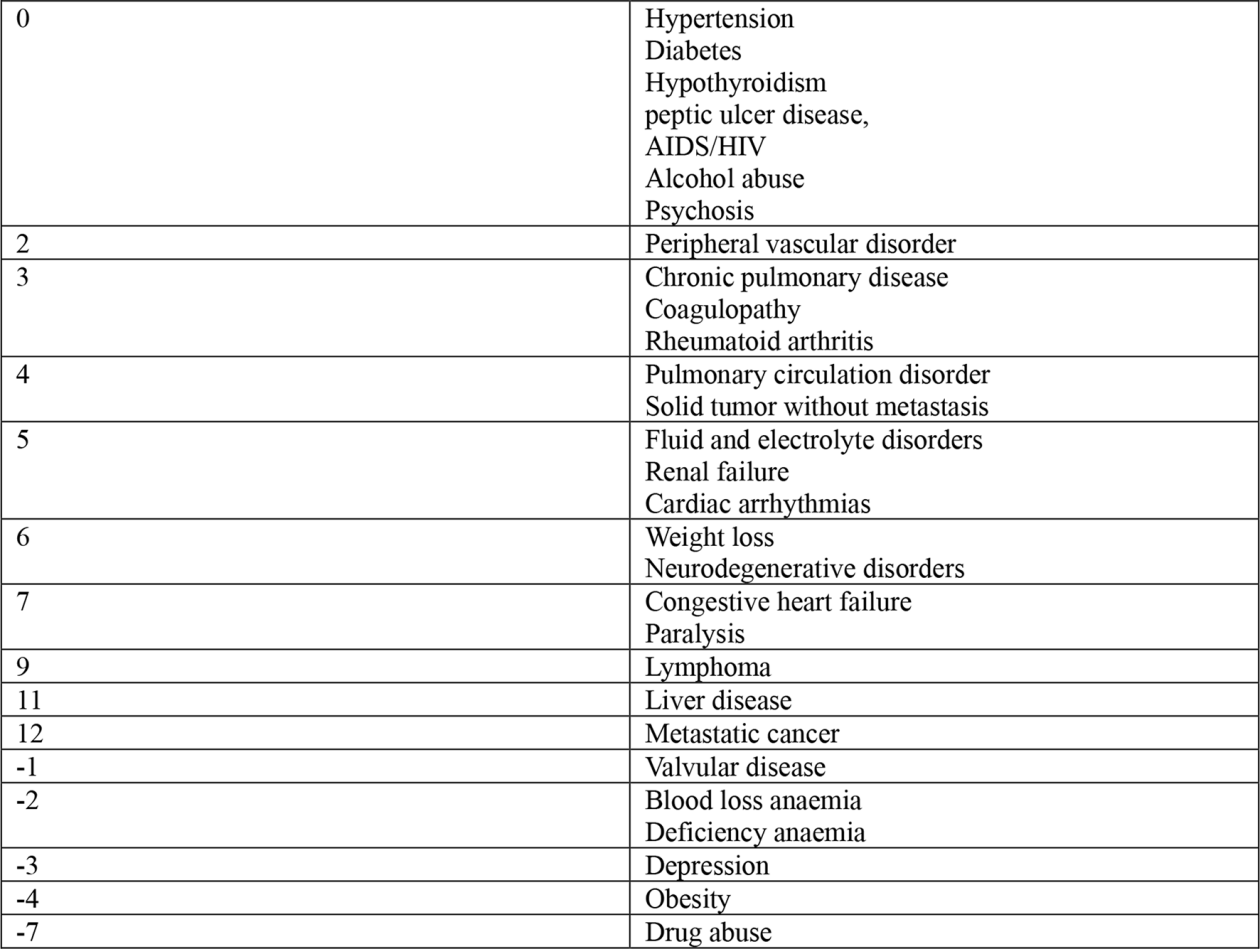
Elixhauser-Comorbidity-Index. Including 30 categories of comorbid condition. It was developed using administrative data for the prediction of length of stay, hospital charges and in-hospital mortality. Modified after [13]

**Fig. 3:**
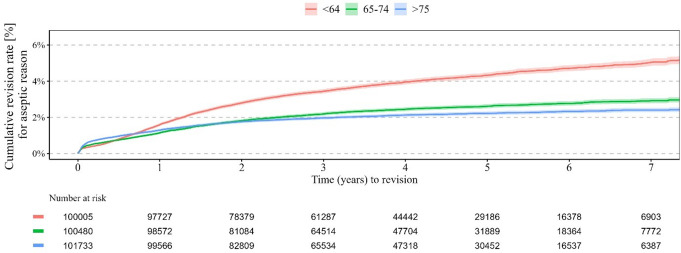
Cumulative aseptic revision rates according to the age

Patients aged between 64 and 75 years demonstrated a rate of aseptic revision of 1.1% after one year, 2.2% after three years and 2.9% after seven years. After a year, 1.3% of patients older than 74 years required a revision due to an aseptic reason. For an aseptic reason after three years 2.0% and after seven years 2.4%, respectively, required revision. An aseptic rate of 1.6% after one year, 3.4% after three years, and 5% after seven years was determined in patients younger than 65 years. Patients younger than 65 years demonstrated thereby a significant increased aseptic rate of revision compared to patients between 65 and 74 years and patients older than 75 years (*p* < 0.0001) (Table 3; Fig. 2 and 3).

**Table 3:** Cumulative rate of aseptic revisions according to the different age with corresponding 95%-Confidence interval

	1 Month	3 Months	6 Months	1 Year	3 Year	5 Years	7 Years
Age ≤ 64 in % (95%-Confidence Interval)	0.3 (0.3,0.3)	0.5 (0.4,0.5)	0.8 (0.7,0.9)	1.6 (1.5,1.7)	3.4 (3.3,3.6)	4.3 (4.2,4.5)	5 (4.9,5.3)
Age 65–74 in % (95%-Confidence Interval)	0.4 (0.3,0.4)	0.6 (0.5,0.6)	0.8 (0.7,0.8)	1.1 (1.1,1.2)	2.2 (2.1,2.3)	2.6 (2.5,2.7)	2.9 (2.8,3.1)
Age ≥ 75% (95%-Confidence Interval)	0.5 (0.5,0.6)	0.8 (0.7,0.8)	1 (0.9,1.0)	1.3 (1.2,1.4)	2.0 (1.9,2.1)	2.2 (2.1,2.3)	2.4 (2.3,2.5)

Gender or weight were not demonstrated as protective or risk factor regardless the patients’ age in constrained TKA (age ≤ 64 (HR = 0,98; *p* > 0,9); age 65–74 (HR = 1,23; *p* = 0,4); age ≥ 75 (HR = 0,82; *p* = 0,4)). Neither TKA implantation volume nor various Elixhauser scores could be identified as a risk or preventive factors (Table 4).

**Table 4:** Hazard ratio (HR) for aseptic revisions in constrained TKA according to the age

Characteristic	Age ≤ 64			Age 65–74			Age ≥ 75		
	HR	95% CI	*p*-value	HR	95% CI	*p*-value	HR	95% CI	*p*-value
BMI (kg/m²)									
Underweight (< 18,5)	0	0.00, 0.00	< 0.001	0	0.00, 0.00	< 0.001	0	0.00, 0.00	< 0.001
Normal (18,5–24,9)	1,38	0.51, 3.79	0,5	1,08	0.43, 2.74	0,9	1,4	0.87, 2.23	0,2
Pre-obesity (25–29,9)	1,29	0.59, 2.82	0,5	1,35	0.70, 2.61	0,4	1,42	0.93, 2.18	0,11
Obesity grade I (30–34,9)	1,05	0.47, 2.38	> 0.9	1,23	0.64, 2.38	0,5	0,77	0.42, 1.40	0,4
Obesity grade II (35–39,9)	1,52	0.71, 3.27	0,3	1,36	0.63, 2.94	0,4	0,56	0.20, 1.61	0,3
Obesity grade III (> 40)	1,07	0.47, 2.43	0,9	0,2	0.03, 1.50	0,12	1,09	0.26, 4.53	> 0.9
Male	0,98	0.56, 1.72	> 0.9	1,23	0.73, 2.06	0,4	0,82	0.53, 1.28	0,4
TKA implantation volume									
101–250	0,91	0.49, 1.67	0,8	1,14	0.66, 1.99	0,6	1,06	0.71, 1.59	0,8
> 250	0,74	0.39, 1.42	0,4	0,73	0.39, 1.37	0,3	1,03	0.67, 1.58	> 0.9
Elixhauser score									
0	1,4	0.72, 2.71	0,3	0,78	0.43, 1.42	0,4	0,58	0.33, 1.04	0,066
1–4	1,57	0.68, 3.63	0,3	0,78	0.36, 1.72	0,5	0,77	0.38, 1.55	0,5
≥ 5	1,36	0.60, 3.08	0,5	0,84	0.43, 1.65	0,6	0,75	0.43, 1.32	0,3

Within six months postoperative, patients treated with an unconstrained TKA and age younger than 65 years, the Elixhauser score regardless the degree (0: HR = 1,38; *p* = 0,005; 1–4: HR = 1,7; *p* < 0,001; ≥5: HR = 1,75; *p* < 0,001) was identified as risk factor. A TKA volume of 101–250 (HR = 0,66; *p* > 0,001) and a TKA volume > 250 (HR = 0,5; *p* < 0,001) as well as being male (HR = 0,87; *p* = 0,002) were detected as preventive factors for an aseptic revision in unconstrained TKA. After six months postoperative, pre-obese (HR = 0,86; *p* = 0,048), obesity grade I (HR = 0,87; *p* = 0,046) and obesity grade III (HR = 0,77; *p* = 0,005) as well as a TKA volume 101–250 (HR = 0,87; *p* = 0,025) and a TKA volume > 250 per year (HR = 0,72; *p* < 0,001) could be identified as preventive factors.

Within six months postoperative, patients treated with unconstrained TKA and aged between 64 and 75 years, obesity grade II (HR = 1,47; *p* = 0,008), obesity grade III (HR = 2,44; *p* < 0,001) and an Elixhauser score ≥ 5 (HR = 1,54;*p* < 0,001) were identified as risk factors. A TKA volume of 101–250 per year (HR = 0,62; *p* < 0,001) and a TKA volume > 250 (HR = 0,69; *p* < 0,001) could be recorded as a preventive factor. After six months postoperative, an Elixhauser score 1–4 (HR = 1,26;*p* = 0,025) was recorded as a risk factor. Pre-obese (HR = 0,78; *p* = 0,005), obesity grade I (HR = 0,81; *p* = 0,02) as well as a TKA volume > 250 per year (HR = 0,78; *p* = 0,001) were demonstrated preventive factors.

For patients older than 74 years and treated with unconstrained TKA, pre-obese (HR = 1,23;*p* = 0,031), obesity grade I (HR = 1,37; *p* = 0,004), obesity grade II (HR = 1,59; *p* = 0,003), obesity grade III (HR = 2,7; *p* < 0,001), an Elixhauser score 1–4 (HR = 1,63; *p* = 0,001) and an Elixhauser score ≥ 5 (HR = 1,7; *p* < 0,001) were identified as risk factors within six months postoperative. Once more, male sex (HR = 0,88; *p* = 0,018), a TKA volume of 101–250 per year (HR = 0,79; *p* = 0,014) as well as cemented (HR = 0,52; *p* = 0,002) as fixation method were discovered as a preventative factors within six months postoperative in unconstrained TKA. After six months postoperative, obesity grade I (HR = 0,74; *p* = 0,007) was identified as preventive factors (Table 5).

**Table 5:** Hazards ratio (HR) for aseptic revisions in unconstrained TKA according to the age

Characteristic	Age ≤ 64			Age 65–74			Age ≥ 75		
	HR	95% CI	*p*-value	HR	95% CI	*p*-value	HR	95% CI	*p*-value
Cemented	0,89	0.64, 1.23	0,5	0,93	0.59, 1.46	0,7	0,52	0.35, 0.78	0,002
Hybrid	0,92	0.64, 1.33	0,7	0,83	0.50, 1.35	0,4	0,48	0.31, 0.77	0,002
Male	0,87	0.79, 0.95	0,002	0,99	0.89, 1.10	0,8	0,88	0.78, 0.98	0,018
**Within six months postoperative**									
Underweight (< 18,5)	0	0.00, 0.00	< 0.001	2,54	0.35, 18.4	0,4	0	0.00, 0.00	< 0.001
Normal (18,5–24,9)	0,91	0.60, 1.37	0,7	0,91	0.61, 1.35	0,6	1,02	0.79, 1.32	0,9
Pre-obesity (25–29,9)	0,9	0.68, 1.18	0,4	1	0.78, 1.29	> 0.9	1,23	1.02, 1.50	0,031
Obesity grade I (30–34,9)	1,15	0.90, 1.47	0,3	1,1	0.85, 1.41	0,5	1,37	1.10, 1.70	0,004
Obesity grade II (35–39,9)	0,92	0.68, 1.24	0,6	1,47	1.11, 1.96	0,008	1,59	1.17, 2.16	0,003
Obesity grade III (> 40)	1,26	0.93, 1.70	0,14	2,44	1.80, 3.32	< 0.001	2,7	1.76, 4.14	< 0.001
TKA implantation volume annually									
101–250	0,66	0.53, 0.81	< 0.001	0,62	0.50, 0.78	< 0.001	0,79	0.65, 0.95	0,014
> 250	0,5	0.40, 0.62	< 0.001	0,69	0.56, 0.85	< 0.001	0,93	0.78, 1.12	0,4
Elixhauser score									
0	1,38	1.10, 1.72	0,005	0,86	0.68, 1.10	0,2	1,06	0.82, 1.37	0,7
1–4	1,7	1.25, 2.30	< 0.001	1,13	0.84, 1.51	0,4	1,63	1.21, 2.18	0,001
≥ 5	1,75	1.28, 2.39	< 0.001	1,54	1.19, 1.99	< 0.001	1,7	1.32, 2.19	< 0.001
**After six months postoperative**									
Underweight (< 18,5)	0,66	0.09, 4.66	0,7	2,18	0.54, 8.73	0,3	0	0.00, 0.00	< 0.001
Normal (18,5–24,9)	1,05	0.85, 1.30	0,6	1,08	0.85, 1.36	0,5	0,9	0.72, 1.13	0,4
Pre-obesity (25–29,9)	0,86	0.74, 1.00	0,048	0,78	0.65, 0.93	0,005	0,95	0.80, 1.13	0,6
Obesity grade I (30–34,9)	0,87	0.76, 1.00	0,046	0,81	0.69, 0.97	0,02	0,74	0.59, 0.92	0,007
Obesity grade II (35–39,9)	0,88	0.75, 1.03	0,11	0,84	0.67, 1.05	0,12	0,85	0.60, 1.19	0,3
Obesity grade III (> 40)	0,77	0.64, 0.92	0,005	0,9	0.67, 1.20	0,5	0,9	0.49, 1.64	0,7
TKA implantation volume annually									
101–250	0,87	0.77, 0.98	0,025	0,87	0.75, 1.02	0,079	0,97	0.81, 1.16	0,7
> 250	0,72	0.64, 0.81	< 0.001	0,78	0.67, 0.90	0,001	0,88	0.74, 1.05	0,15
Elixhauser score									
0	1,04	0.93, 1.16	0,5	1,09	0.93, 1.28	0,3	0,83	0.67, 1.02	0,079
1–4	0,98	0.82, 1.16	0,8	1,26	1.03, 1.55	0,025	0,9	0.69, 1.19	0,5
≥ 5	1,08	0.91, 1.29	0,4	1,11	0.91, 1.35	0,3	0,85	0.68, 1.06	0,2

For patients treated with UKA and aged younger than 65 years, pre-obesity (HR = 0,76; *p* = 0,003), male gender (HR = 0,81; *p* < 0,001), a UKA volume > 50 per year (HR = 0,62; *p* < 0,001), an Elixhauser score 0 (HR = 0,86; *p* = 0,029) and an Elixhauser score 1–4 (HR = 0,77; *p* = 0,043) demonstrated preventive factors. For patients aged between 65 and 74 years, male (HR = 0,72; *p* < 0,001), a UKA volume > 50 per year (HR = 0,59; 0 = 0,003) were identified as preventive factors. Moreover, cemented UKA (HR = 0,37; *p* < 0,001) was detected as preventive factors within six months postoperative. In patients older than 74 years, once more male (HR = 0,57; *p* < 0,001) was identified as preventive factor. Furthermore, cemented UKA (HR = 0,37; *p* < 0,001) within six months postoperative was preventive for revision (Table 6).

**Table 6:** Hazard ratio (HR) for aseptic revisions in UKA according to the age

Characteristic	Age ≤ 64			Age 65–74			Age ≥ 75		
	HR	95% CI	*p*-value	HR	95% CI	*p*-value	HR	95% CI	*p*-value
BMI (kg/m²)									
Underweight (< 18,5)	1,97	0.65, 5.96	0,2	0	0.00, 0.00	< 0.001	0	0.00, 0.00	< 0.001
Normal (18,5–24,9)	0,92	0.71, 1.17	0,5	0,96	0.67, 1.36	0,8	1,18	0.81, 1.72	0,4
Pre-obesity (25–29,9)	0,76	0.64, 0.91	0,003	1,01	0.80, 1.29	> 0.9	1,13	0.84, 1.53	0,4
Obesity grade I (30–34,9)	0,97	0.82, 1.14	0,7	1,13	0.88, 1.46	0,3	1,28	0.88, 1.85	0,2
Obesity grade II (35–39,9)	0,84	0.68, 1.05	0,12	1,29	0.92, 1.81	0,15	1,18	0.63, 2.22	0,6
Obesity grade III (> 40)	1,06	0.81, 1.38	0,7	1,43	0.81, 2.53	0,2	0,95	0.23, 3.93	> 0.9
Male	0,81	0.72, 0.92	< 0.001	0,72	0.60, 0.87	< 0.001	0,57	0.44, 0.74	< 0.001
UKA implantation volume annually									
11–50	0,98	0.79, 1.22	0,9	0,94	0.67, 1.33	0,7	1,09	0.65, 1.82	0,7
> 50	0,62	0.49, 0.77	< 0.001	0,59	0.42, 0.84	0,003	0,75	0.45, 1.24	0,3
Elixhauser score									
0	0,86	0.75, 0.98	0,029	1	0.78, 1.27	> 0.9	0,85	0.58, 1.24	0,4
1–4	0,77	0.60, 0.99	0,043	0,99	0.70, 1.39	> 0.9	0,95	0.58, 1.56	0,8
≥ 5	1,02	0.81, 1.30	0,8	0,96	0.70, 1.33	0,8	1,01	0.67, 1.52	> 0.9
**Within six months postoperative**									
Cemented	0,73	0.50, 1.06	0,1	0,37	0.26, 0.55	< 0.001	0,37	0.23, 0.59	< 0.001
Hybrid	2,29	0.81, 6.50	0,12	0,72	0.17, 3.06	0,7	0,39	0.05, 2.92	0,4
**After six months postoperative**									
Cemented	1,11	0.90, 1.38	0,3	1	0.71, 1.43	> 0.9	1,03	0.59, 1.79	> 0.9
Hybrid	0,96	0.39, 2.39	> 0.9	0	0.00, 0.00	< 0.001	0,58	0.08, 4.42	0,6

## Discussion

The main finding of this register study on 300,998 patients in the German Arthroplasty Register are the significant higher aseptic revision rates in patients with UKA compared to unconstrained and constrained TKA and the significant increased rate of aseptic revisions for patients younger than 65 years compared to patients older than 65 years. In constrained TKA, no risk or preventive factors in the three different age groups could be elucidated. An increased Elixhauser score was a risk factor in unconstrained TKA across all age groups. Male and a high TKA or UKA volume were identified as preventive factor.

### Rate of aseptic revisions according to the age

Aseptic reasons are a major cause of surgical revision after primary knee arthroplasty [14, 15]. Aseptic revision rates vary throughout researches and procedures [9, 15–17]. An analysis of the Danish knee arthroplasty register from 1997 to 2017 reported higher revision risk and lower mortality risk for UKA vs. TKA at all time points [18]. When comparing TKA patients with UKA patients, the revision risk for the majority of present patients dropped over the previous 20 years from a 3-year HR of approximately 5 to an HR of 1.5 [18]. Moreover, revision surgery is anticipated to be necessary for a higher percentage of younger individuals as the number of younger patients undergoing TKA rises [8]. Young patients at the time of primary TKA have been linked to increased rates of reoperation and failure [19]. Mc Calden et al. investigated the patient outcome and the revision rate in different age groups in TKA, however no subdivision into unconstrained and constrained TKA was carried out. For this purpose, the cohort was divided into three age groups: patients younger than 55 years, patients between 55 and 70 years and patients older than 70 years. A higher revision rate was found in patients younger than 55 years. The overall revision rate was 5.9% for patients younger than 55 years, 3.1% for patients between 55 and 70 years and 2.1% for patients older than 70 years [11]. Charette et al. also reported a higher rate of revision in young patients [20]. In the present analysis the cohort was divided into patients younger than 65 years, patients aged between 65 and 74 years and patients older than 74 years. Our analysis demonstrated comparable results with a significant increased aseptic rate of revision in patients younger than 65 years compared to patients aged between 65 and 74 years and patients older than 75 years (*p* < 0.0001). Moreover, young patients under 55 years were more likely than patients older than 55 years to undergo early revision of TKA within two years following primary TKA (52.5% vs. 29.0%) [21]. Even though TKA is an excellent therapy option for younger patients with osteoarthritis of the knee, it is crucial to inform these patients about the lower survival rate associated with knee arthroplasty. Compared to older patient groups, younger patients had poorer TKA survivability but equal or possibly superior clinical results after primary TKA [11]. Early-life history of knee prosthesis implantation is associated with a higher likelihood of co-morbidities and potentially poorer health characteristics [5, 19, 21]. According to Keeney et al. a higher body mass index and lower activity levels are found among younger patients undergoing primary knee arthroplasty [22]. One the one hand, this leads to a higher revision rate due to septic reasons because of an increased risk for infection. On the other hand, an increased risk of aseptic mechanical failure due to the patients’ weight could be observed [13, 26]. Moreover, in younger patients, sustained high activity levels are unlikely to be a main cause for aseptic revision of primary knee arthroplasties [22]. Comparable results could be achieved in the case of a UKA. The risk of revision was once more highest in the youngest age group from 46 to 50 years with 40.4% and decreased sequentially until the oldest age group from 86 to 90 years which accounted 3.7% [23]. Younger patients have a higher risk of revision due to progression of osteoarthritis of the knee or aseptic loosening [24, 25]. In UKA, younger age is thought to correlate with increased activity, which may accelerate wear, aseptic loosening or progression of osteoarthritis [26]. In addition, younger patients may have greater expectations of their post-operative function and activity level. Therefore, further surgery will be discussed if pain returns [26].

### Influencing factors

In the present investigation, the risk and preventive factors for UKA as well as constrained and unconstrained TKA were analyzed according to age groups.

In constrained TKA, neither weight, gender, Elixhauser score or TKA volume could be identified as a risk or preventive factor in all three age groups. Studies examining influencing factors in constrained and unconstrained TKA as well as UKA with regard to the individual age groups are rare. An analysis by the New Zealand Joint Registry from January 1999 to Dezember 2016 for risk factors of TKA revision demonstrated that patients between 45 and 50 years of age have the worst 10-year implant survival rate of 91.5%, and the implantat survival rate gradually improved to 99.2% in patients between 90 and 95 years of age [27]. In our study a time split after six months was conducted to reduce the violation of confounding variables when assuming constant proportional risks.

In unconstrained TKA, an Elixhauser score of 1–4 (HR = 1,7; *p* < 0,001) and an Elixhauser score ≥ 5 (HR = 1,75; *p* < 0,001) for patients younger than 65 years as well as an Elixhauser score 1–4 (HR = 1,26;*p* = 0,025) and an Elixhauser score ≥ 5 (HR = 1,54;*p* < 0,001) in patients aged between 65 and 74 years were detected as risk factors. Moreover, an Elixhauser score 1–4 (HR = 1,63; *p* = 0,001) and an Elixhauser score ≥ 5 (HR = 1,7;*p* < 0,001) in patients older than 74 years were identified as risk factors. An analysis by the New Zealand Joint Registry from January 1999 to Dezember 2016 for risk factors of TKA revision identified the highest risk of revision with 24.5% in patients with ASA grade 3 and 4 aged between 46 and 50 years, compared to 18.9% in ASA grade 1 patients in the same age group (*p* < 0.001) [27]. In contrast to our study, where male was identified as a preventive factor for patients younger than 65 years (HR = 0,87; *p* = 0,002) and older than 74 years (HR = 0,88; *p* = 0,018), the analysis of the New Zealand Joint register detected male as a risk factor for revision in TKA [27]. Mikkelsen et al. elucidated, that cementless TKAs had higher revision risks than cemented TKAs (HR 1.7, CI 1.4–1.9) [18]. Moreover, Fleischman et al. reported significantly reduced risk for mechanical failure with cemented TKA by 58.9% (*p* = 0,0002) with increasing age for each additional decade of life [28]. In the present study, the use of a cemented system in unconstrained TKA (HR = 0,52; *p* = 0,002) was also identified as a preventive factor in patients older than 74 years. An analyses of Nordic Arthroplasty Register Association between 2000 and 2016 identified uncemented TKA with increased risk of revision compared with the cemented TKA (HR = 1.3) [29]. Namba et al. identified for TKA afro-american patients (HR = 1.73), diabetes (HR = 1.21), a volume of performed TKA under 50 (HR = 1.11), as well as again cementless knee arthroplasty (HR = 1.28) as risk factors for aseptic revision [15]. Gelderman et al. reported post-operative dissatisfaction being more common in younger patients (*<* 55 years) and younger patients having the double risk to undergo early revision than older patients (*>* 60–75 years) [30]. However, in literature patient-related preventive factors according to the patients’ age and type of knee prothesis are rarely discussed. Namba et al. mentioned a 37% decreased risk of revision by same day bilateral procedures in TKA [15]. Moreover, an BMI > 35 kg/m² vs. BMI < 30 kg/m² had a lower risk for aseptic revision (HR = 0.78) [15]. In comparison, in the present study, pre-obese (HR = 0,86; *p* = 0,048), obesity grade I (HR = 0,87; *p* = 0,046) and obesity grade III (HR = 0,77; *p* = 0,005) could be identified as preventive factor after six months postoperative in patients under 64 years in unconstrained TKA. However, in the patients aged between 64 and 75 years, obesity grade II (HR = 1,47; *p* = 0,008) and obesity grade III (HR = 2,44; *p* < 0,001) were reported as risk factors for unconstrained TKA within six months postoperative, whereas pre-obese (HR = 0,78; *p* = 0,005) and obesity grade I (HR = 0,81; *p* = 0,02) were identified as preventive factors after 6 months postoperative. In patients older than 74 years, pre-obese (HR = 1,23;*p* = 0,031), obesity grade I (HR = 1,37; *p* = 0,004), obesity grade II (HR = 1,59; *p* = 0,003) and obesity grade III (HR = 2,7; *p* < 0,001) were detected as risk factors within six months postoperative, whereas after six months postoperative, obesity grade I (HR = 0,74; *p* = 0,007) could be found as preventive factor. Thus, the weight’s role as a preventive or risk factor in unconstrained TKA varies depending on the period of time and the patient’s age. Boyer et al. mentioned no influence of Diabetes mellitus status (*p* = 0.9657), BMI (*p* = 0.4517) or gender (*p* = 0.88) on survival for aseptic loosening in TKA [31]. Moreover, in the present study, for patients younger than 65 years, a TKA volume > 250 per year (HR = 0,72; *p* < 0,001) and a TKA volume of 101–250 per year (HR = 0,66; *p* > 0,001) after six months postoperative. Furthermore, a TKA volume > 250 per year (HR = 0,5; *p* < 0,001) within six months postoperative were detected as preventive factors for an aseptic revision in unconstrained TKA. For patients aged between 64 and 75 years, a TKA volume of 101–250 per year (HR = 0,62; *p* < 0,001) and a TKA volume > 250 per year (HR = 0,69; *p* < 0,001) within six months postoperative as well as a TKA volume of > 250 per year (HR = 0,78; *p* = 0,001) after six months postoperative were also identified as preventive factors. For patients older than 74 years, a TKA volume of 101–250 per year (HR = 0,79; *p* = 0,014) was recorded as preventive factors within six months postoperative. Moreover, Badaway et al. reported increasingly better results with increasing annual hospital volume [32].

In case of UKA, cemented prothesis were analyzed as preventive factor in patients aged between 65 and 74 years (HR = 0,37; *p* < 0,001) and in patients older than 74 years (HR = 0,37; *p* < 0,001) within six months postoperative. The identification of patient comorbidities and prosthesis specific influencing factors for aseptic revision of UKA varies among the literature. However, in literature patient-related and prothesis specific influencing factors with regard to the patient age are rarely discussed. Mikkelsen et al. elucidated, that revision risk was lower for cementless UKA compared with cemented UKA (HR 0.6, CI 0.5–78) [18]. Moreover, Tay et al. reported a higher risk of aseptic revision for cemented mobile-bearing UKA compared with cemented fixed-bearing UKA (HR = 1.9, 95% CI = 1.1–3.2; *p* = 0.03) [33]. Additionally, in our investigation, a UKA volume > 50 per year (HR = 0,62; *p* < 0,001) in patients younger than 65 years or patients aged between 65 and 74 years (HR = 0,59; *p* = 0,003) were preventive factors. In comparison, an analyses of the Dutch arthroplasty register from 2007 to 2016, hospitals with higher TKA volume were more likely to use UKA and hospitals with a higher absolute or proportional UKA volume could improve survival [34]. Hospitals with an absolute volume of performed UKA between 22 and 36 per year had a higher risk for revision (HR = 1.04), whereas a UKA volume between 36 and 58 annually (HR = 0.96) and a UKA volume more than 58 (HR = 0.74) annually leaded to a lower risk of aseptic revision [35]. This is comparable to our findings that a high volume of UKA implantations can lead to a satisfactory functional outcome. Younger individuals had a higher likelihood of revision than older patients (≥ 70 years). In UKA, patients under 60 years old demonstrated a 1.9-fold increase in aseptic revision risk, while those between 60 and 69 years old presented a 1.6-fold rise in aseptic revision risk (< 60 years: HR = 1.9, 95% CI = 1.2-3.0; 60–69 years: HR = 1.6, 95% CI = 1.0-2.4; *p* < 0.05) [33]. Additionally, a higher cumulative frequency of revision for aseptic loosening in UKA in younger patients was observed (3.2% vs. 2.7% for ≥ 70 years; *p* < 0.05) [33]. Moreover, in the present analyses in all three age categories, male was found as a preventative factor (< 65: HR = 0,81; *p* < 0,001; 65–74: HR = 0,72; *p* < 0,00; >74: HR = 0,57; *p* < 0,001). Additionally, pre-obese (HR = 0,76; *p* = 0,003), an Elixhauser score 0 (HR = 0,86; *p* = 0,029) and an Elixhauser score 1–4 (HR = 0,77; *p* = 0,043) could be identified as a preventive factors for aseptic revision of UKA in patients younger than 65 years. The identification of patient co-morbidities as risk factors for aseptic revision of UKA varies among the literature. However, in literature patient-related influencing factors with regard to the patient age are rarely discussed. Tay et al. reported a 1.2- to 1.5-fold higher lifetime risk of revision for UKA for females compared to males across all age groups. Women have a lower mortality rate than men in all age groups. Therefore, it can be assumed to live longer and be exposed to a higher revision rates [23, 36]. Boyer et al. mentioned no influence of Diabetes status (*p* = 0.1186) or BMI (*p* = 0.4561) on survival for aseptic loosening in UKA. However, in contrast to the present investigation, a substantial increase in risk in UKA was discovered for females (*p* = 0.0175) [31].

### Limitations

Despite multiple advantages of the German Arthroplasty Registry, several limitations of the present study are worth to be mentioned. The accurate coding of procedures and surgeon registration are essential for the quality of data in this registry. The included patient data were cross-validated using insurance data in order to reduce this impact and constraint. Due to different indications of the investigated implants different quantities were reported. Kaplan-Meier-estimates, the corrected multiple Log-Rank-Test as well as a time split after six months for the calculation of hazard ratio were used to reduce this limitation. An additional limitation pertains to the length of the registry’s existence, as it presently precludes the investigation of follow-ups lasting more than seven years. Inaccurate or inadequate coding is another potential cofounder. The Elixhauser Comorbidity score was computed using the comorbidities recorded during the initial hospital stay after primary implantation. However, not all comorbidities are evaluated using the Elixhauser Comorbidity Index. Moreover, the present analysis’s informative value is diminished by the incomprehensibility of the stem lengths for shaft-anchored prosthesis based on registry data. Another limitation is the fact that the degree of coupling cannot be detailed for constraint prostheses. An additional constraint pertains to the analysis of the individual revisions’ causes solely to the type of care, without considering the varying age groups included within that category.

## Conclusion

Patients younger than 65 years are more likely to undergo aseptic revisions than patients older than 65 years. An increased Elixhauser score demonstrated a risk factor for aseptic failure, whereas male gender as well as a high volume of performed UKA for patients younger than 65 and aged between 65 and 74 years as well as a high volume of performed TKA regardless the age could be identified as preventive factors. Therefore, affected patients should be informed and prepared preoperatively according to their individual risk and preventive factors. Consequently, individual risk factors such as BMI may be optimized preoperatively and preventive factors can be improved by selecting the appropriate medical facility.

## Data Availability

Data available on request.
